# Retinal layers prognosticate cognitive progression independent of relapse activity in multiple sclerosis

**DOI:** 10.1007/s00415-026-14036-0

**Published:** 2026-08-06

**Authors:** Nuria Cerdá-Fuertes, Shaumya Sankar, Silvan Pless, Marc Stoessel, Shaumiya Sellathurai, Kean Schoenholzer, Federico Burguet Villena, Alessandro Cagol, Lisa Hofer, Anastasios Demirtzoglou, Bettina Fischer-Barnicol, Pasquale Calabrese, Pascal Benkert, Jannis Müller, Marcus D’Souza, Konstantin Gugleta, Tobias Derfuss, Cristina Granziera, Ludwig Kappos, Jens Kuhle, Athina Papadopoulou

**Affiliations:** 1 Translational Imaging in Neurology (ThINK) Basel, Department of Medicine and Biomedical Engineering, University Hospital Basel and University of Basel, Basel, Switzerland; 2 Research Center for Clinical Neuroimmunology and Neuroscience, University of Basel, Basel, Switzerland; 3 Neurologic Clinic and Policlinic, Departments of Medicine, Clinical Research and Biomedical Engineering, University Hospital Basel and University of Basel, Basel, Switzerland; 4 Department of Psychology and Interdisciplinary Platform Psychiatry and Psychology, Division of Molecular and Cognitive Neuroscience, University of Basel, Basel, Switzerland; 5 Department of Clinical Research, University of Basel, Basel, Switzerland; 6 University Eye Clinic Basel, University Hospital Basel and University of Basel, Basel, Switzerland; 7 Dipartimento di Scienze della Salute, Università degli Studi di Genova, Genoa, Italy; 8 Department of Neurology, Inselspital, University Hospital Bern, University of Bern, Bern, Switzerland

**Keywords:** OCT, pRNFL, mGCIPL, mINL, BICAMS, SDMT, VLMT, PIRA

## Abstract

**Background:**

Optical coherence tomography (OCT) quantifies retinal neuroaxonal loss related to neurodegeneration in people with multiple sclerosis (pwMS) and is associated with physical and cognitive disability. However, no data exist on the relationship between OCT metrics and cognitive progression independent of relapse activity (cognitive PIRA).

**Objective:**

To assess if OCT can prognosticate cognitive PIRA events in pwMS.

**Methods:**

Prospective study with baseline OCT (peripapillary retinal nerve fiber- (pRNFL), macular ganglion cell-inner plexiform- (mGCIPL) and inner nuclear layers (mINL)) and brief international cognitive assessment for MS (BICAMS) at yearly visits. Cognitive PIRA in each test was defined as more than 10% decrease compared to previous visit (reference) and confirmed 12 months later (confirmation visit), without relapses 90 days before or 30 days after the event and confirmation visits. We employed Cox regression models adjusting for age, baseline cognitive test, education, and treatment.

**Results:**

98 pwMS followed for a median of 5 years (63% female, 81% relapsing–remitting, age: 50.5 ± 11.6 years, median EDSS: 3.0). Each 1 µm increase in pRNFL thickness was associated with a 6% lower risk of Symbol Digit Modalities Test (SDMT) PIRA events (Hazard ratio (HR) = 0.94, *p* = 0.027). Each 1 µm increase in mGCIPL thickness was associated with a 6% lower risk of Verbal Learning and Memory Test (VLMT) PIRA (HR = 0.94, *p* = 0.040). Each 1 μm increase in mINL thickness was associated with 20% lower risk of VLMT-PIRA (HR = 0.80, *p* = 0.030) and 50% *higher* risk of SDMT-PIRA events (HR = 1.50, *p* = 0.049).

**Conclusion:**

Retinal layers can be sensitive, patient-friendly prognostic markers of cognitive PIRA in pwMS.

**Supplementary Information:**

The online version contains supplementary material available at https://doi.org/10.1007/s00415-026-14036-0.

## Introduction

Neuronal and axonal loss represent a major pathological substrate underlying disability in multiple sclerosis (MS)[1]. Optical coherence tomography (OCT) is a noninvasive, quick, patient-friendly examination that can provide markers of neuroaxonal loss. Specifically, the macular ganglion cell layer and peripapillary retinal nerve fiber layer (pRNFL)—which contain neurons and axons of the visual pathway, respectively—are associated with gray matter integrity on brain magnetic resonance imaging (MRI) [2], as well as physical and cognitive disability in people with MS (pwMS) [3, 4]. Moreover, OCT measures were associated with progression independent of relapse activity (PIRA) in prior studies [5–7], suggesting that retinal atrophy reflects relevant neurodegenerative processes in the central nervous system (CNS). However, PIRA is typically based on the expanded disability status scale (EDSS) and thus primarily reflects physical disability [8]. More recently, the concept of *cognitive* PIRA has been proposed [9].

Cognitive impairment is a prevalent (34–65%) and disabling manifestation in pwMS, even at early stages of the disease and across all phenotypes. It can develop insidiously and progress gradually or decline abruptly during relapses [10]. Previous studies showed longitudinal associations between OCT measures and cognitive impairment [11–13]. Moreover, a threshold of pRNFL thickness ≤ 88 µm has been associated with an increased risk of cognitive decline [12, 13]. In another study, both macular ganglion cell-inner plexiform layer (mGCIPL) and pRNFL at baseline (BL), as well as their annual thinning rates, predicted physical and cognitive disability progression [11]. However, these studies did not further differentiate cognitive decline due to PIRA versus relapse-associated worsening. Thus, the value of OCT measures on prognosticating the risk of cognitive PIRA has not been investigated yet.

Our primary aim was to assess the value of retinal layer thickness (as quantified by OCT) to prognosticate events of cognitive PIRA in pwMS. Moreover, we aimed at investigating the relationship between OCT measures and risk of cognitive PIRMA events (progression independent of relapses *and* MRI activity) as well as of events with purely cognitive PIRA, not accompanied by worsening in physical disability (EDSS).

## Methods

### Study design and participants

**Fig. 1:**
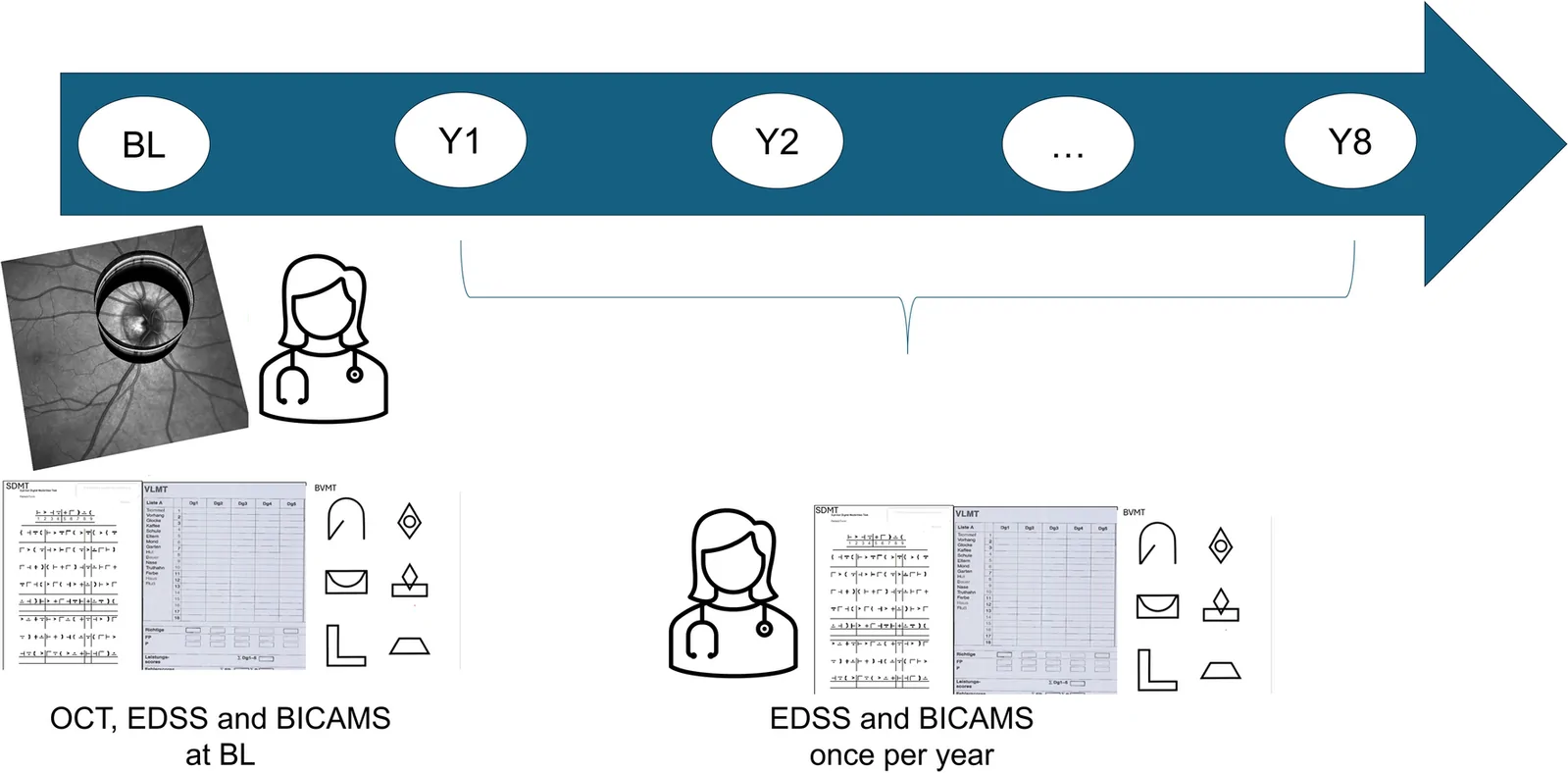
Timeline of the longitudinal study illustrating baseline and follow-up visits, the assessments conducted and the number of patients at each visit. Abbreviations: *BICAMS * Brief International Cognitive Assessment for MS, *BL * baseline, *EDSS * expanded disability status scale, , *n* number of patients, *OCT *optical coherence tomography, *Y* year

We performed a prospective study with OCT at BL and cognitive testing as well as neurological examination (including the Neurostatus EDSS) at BL and yearly visits (Fig. 1, flowchart). The cross-sectional analysis at BL has been previously described [3], pwMS were recruited from a local cohort in the MS Center at the University Hospital of Basel (UHB) between 2016 and 2017 and followed up until 2024, with variable follow-up duration. No formal sample size calculation was performed for the longitudinal follow-up; the study size was determined by all eligible participants recruited at BL. The inclusion criteria were: (i) age ≥ 18 years, (ii) eligibility for OCT (i.e., ability to fixate with each eye) and MRI (e.g., no claustrophobia), (iii) MS diagnosis according to the 2010 revised McDonald criteria. The exclusion criteria were: (i) serious ophthalmological comorbidity, (e.g., glaucoma), (ii) refractive errors > 6 diopter, and (iii) bilateral optic neuritis (ON) history, as assessed by experienced neurologists [3].

This manuscript is reported in accordance with the STROBE guidelines for cohort studies.

### OCT

OCT was performed at BL, on a Heidelberg Engineering Spectralis device (Heidelberg, Germany), in a dark room, without pupil dilation. The following OCT metrics were assessed on both eyes: the mean pRNFL thickness, the thickness of the mGCIPL, as well as of the macular inner nuclear layer (mINL).

OCT quality control was performed applying the OSCAR-IB criteria [14]. A total of 21 eyes were excluded from macular layers’ analysis (13 due to quality reasons and 8 due to incidental findings), while 5 eyes were excluded from the pRNFL analysis (2 for quality reasons and 3 due to incidental findings). Eyes with history of ON (*n* = 35) were excluded, since the local damage would be a confounder to our analysis. For patients with unilateral ON, we included only the non-affected eye. Furthermore, we excluded eyes with possible local damage (subclinical optic nerve lesion) by calculating the inter-eye asymmetry, using previously described thresholds: for pRNFL ≥ 5 µm and for mGCIPL ≥ 4 µm and excluding the eye with the lower thickness: in case of asymmetry only in pRNFL, we excluded only pRNFL of the worst eye, but not macular layers, in case of asymmetry in mGCIPL, we excluded also mINL of the worse eye, but not pRNFL thickness [15–17]. For those patients without history of ON or ocular asymmetry, we took the average values of both eyes.

Further details regarding the OCT protocol are previously described [3] and also shown as supplementary table (Online Resource 1), according to the APOSTEL recommendations [18].

### Cognitive testing

All pwMS underwent cognitive evaluation performed by trained test raters using the Brief International Cognitive Assessment for MS (BICAMS) [19] at BL and annual follow-up visits. BICAMS comprises three tests: the Symbol Digit Modalities Test (SDMT, oral version) to evaluate information processing speed, the German version of the Rey Auditory Verbal Learning Test, known as “Verbaler Lern- und Merkfähigkeitstest” (VLMT), to evaluate verbal memory, and the Brief Visuospatial Memory Test (BVMT), to evaluate visual memory. While SDMT and VLMT were performed until last visit (maximum follow-up: 8 years), BVMT was only performed until year 4. Both VLMT and BVMT had multiple versions (3 versions for VLMT and 6 versions for BVMT), which were alternated each year to reduce potential learning effects. One patient did no undergo any BVMT testing over the entire follow-up.

A cognitive PIRA event was defined in each cognitive test as a > 10% decrease compared to the previous visit (roving reference), confirmed at the following 12-monthly visit with the same or a lower score (confirmation), in absence of relapses within the 90 days before and 30 days after the event- and confirmation visit, as previously suggested [8]. A second event in the same cognitive test could occur one year later after the confirmation date. We decided to use the roving reference approach, as it has been shown to increase sensitivity to detect progression/worsening events in pwMS [20].

Cognitive PIRMA was defined as a PIRA event without any new or enlarging lesions on fluid-attenuated inversion recovery (FLAIR) sequences of brain MRI, between reference and confirmation visit.

Finally, “purely cognitive” PIRA events fulfilled the above PIRA definition but additionally were not accompanied by a relevant increase in the EDSS compared to the previous visit (reference visit) that was confirmed after 12 months. The criteria for a relevant EDSS increase were as follows: an increase of ≥ 1.5 points if the BL EDSS is 0, an increase of ≥ 1 point if the BL EDSS is between 1.0 and 5.0, or an increase of ≥ 0.5 points if the BL EDSS is > 5.0 [8].

### MRI

To adjust for brain imaging measures of inflammatory and neurodegenerative damage in the analysis, MRI data from BL were included. For most pwMS, the MRI was performed at the same day as cognitive testing, for 15 patients, within a maximum period of 17 days and for one patient 80 days before, while three patients had no MRI. All MRI examinations were performed in the Clinic of Radiology at the UHB, in a 3 Tesla Scanner (Skyra, Siemens, Erlangen, Germany). The MRI-protocol at BL included a 3D T1 weighted magnetization prepared rapid gradient-echo (MPRAGE) and a 3D FLAIR sequence, as previously described [3]. MS lesions were segmented on the FLAIR sequence with the deep-learning based tool MD-GRU[21] and manually corrected where necessary by an experienced rater, to obtain total lesion volume (TLV). Brain volumetric measurements were obtained with FreeSurfer (v. 6.0.0). FreeSurfer reconstructions were manually checked and corrected, to obtain gray matter volume (GMV) and total intracranial volume. Three MRIs were excluded from volumetric analysis due to poor image quality.

Moreover, MRI data was used to identify PIRMA events. FLAIR images of patients with PIRA obtained between the reference and confirmation visits were reviewed to assess the presence of new or enlarging lesions.

### Statistics

The distribution of continuous variables was assessed visually using Q–Q plots.

To assess the relationship between OCT metrics at BL and the risk of subsequent (i) cognitive PIRA, (ii) cognitive PIRMA, and (iii) purely cognitive PIRA events in each cognitive test, we employed Cox regression models. These models were adjusted for: age at BL, years of education, BL score of each cognitive test, and disease-modifying therapy (DMT). We used the “dominant” DMT during observation time and it was classified in untreated, low/medium efficacy (injectable platform therapies, teriflunomide or dimethyl fumarate) and high-efficacy DMT (fingolimod, cladribine, natalizumab, and anti-CD20 monoclonal antibodies). Notably, we did not correct for annualized relapse rate, as the definition of cognitive PIRA already excludes clinical activity 90 days before and 30 days after the event. Sex was not included as a covariate, as there were no significant differences in sex distribution between groups (PIRA/no PIRA in each test). In case of missing baseline cognitive score (1 from BVMT and 2 for VLMT), the score from year 1 was used.

Based on the results of our main analysis, we further explored the associations between retinal layer thickness and the risk of cognitive PIRA in different cognitive tests, adjusting for measures of inflammatory (lesional) versus neurodegenerative (GMV) damage in the brain. Thus, we included first TLV and then GMV (adjusting also for total intracranial volume), in the Cox regression models that had shown associations between OCT measure at BL and cognitive PIRA events. Note that we did not include TLV and GMV in the same model to avoid collinearity problems and facilitate interpretation.

We did not correct the p values for multiple comparison, because the analysis was mainly exploratory. As such, the presentation of *p* values is intended to indicate magnitude of effects rather than statistical significance.

All available data for each participant were included in the analysis. Missing values were not considered in specific statistical tests. To assess the potential impact of loss to follow-up, a sensitivity analysis including only data until visit 5 was performed.

All statistical analyses were performed using R (20) version 4.2.5 with packages ggplot2, pastecs, car, effects, multcomp, stats, WRSS, sjPlot, dplyr, reshape2, nnet, survival, survminer, and logistf.

## Results

### Baseline characteristics

Overall, 98 pwMS were included in this study. Their BL characteristics are summarized in Table 1.

**Table 1:** Baseline characteristics of pwMS included in the study

*N*	98
Female, *n* (%)	62 (63%)
Age in years, mean ± SD	50.5 ± 11.6
Years of education, mean ± SD	14.1 ± 3.3
EDSS, median (IQR)	3.0 (2.0–3.5)
Disease form, PMS/RRMS, n (%)	19 (19.4%)/79 (80.6%)
Disease duration in years, mean ± SD	19 ± 10
Disease activity, yes *n* (%)	
Relapses during last year before BL	0 (0%)
Relapses within 3 months after BL	0 (0%)
DMT, *n* (%)^a^	
Untreated	24 (24.5%)
Low/medium efficacy	19 (19.4%)
High efficacy	55 (56.1%)
OCT metrics	
Average pRNFL thickness in µm Mean ± SD	93.0 ± 14.4
Average mGCIPL thickness in µm Mean ± SD	66.3 ± 8.1
Average mINL thickness in µm Mean ± SD	35.0 ± 2.3
Cognitive performance	
SDMT score, mean ± SD (range)^b^	54.0 ± 15.1 (16–110)
VLMT score, mean ± SD (range)^c^	57.6 ± 9.1 (25–74)
BVMT score, median (IQR) (range)^d^	27 (21–30) (10–36)
N of patients with impaired BICAMS test at baseline^e^	
SDMT, *n* (%)	13 (13.3%)
VLMT, *n* (%)	9 (9.3%)
BVMT, *n* (%)	8 (8.2%)
Cerebral MRI AT BL	
Total lesion volume in ml, median (IQR)	6.9 (1.8–15.9)
Total gray matter volume in ml, mean ± SD	564.9 ± 54.5

^a^Disease-modifying therapies are divided into untreated, low/medium efficacy (injectable platform therapies, teriflunomide or dimethyl fumarate) and high-efficacy DMT (fingolimod, cladribine, natalizumab and anti-CD20 monoclonal antibodies). ^b^maximum possible score for SDMT: 110

^c^maximum possible score for VLMT: 75

^d^maximum possible score for BVMT:36

^e^A cognitive test was considered impaired for a patient if he or she scored at least 1.5 standard deviation (SD) below the average of the healthy controls from our previous publication [3]. Note that normally distributed variables are presented as mean± standard deviation and non-normally distributed as median and interquartile range

Abbreviations: *BICAMS * Brief international cognitive assessment for MS, *BL* baseline, *BVMT* brief visuospatial Memory test, *DMT* disease-modifying therapy, *EDSS* expanded disability status scale, *FLAIR* fluid-attenuated inversion recovery, *mGCIPL* macular ganglion cell-inner plexiform layers, *mINL* macular inner nuclear layer, *OCT* optical coherence tomography, *PMS* progressive Multiple Sclerosis, *pRNFL* peripapillary retinal nerve fiber layer, *RRMS* relapsing–remitting Multiple Sclerosis, *SDMT* symbol-digit modalities test, *VLMT* Rey auditory verbal learning Test

### Cognition over follow-up

PwMS were followed up for a median of 5 years (IQR 4–6 years). Eighty-four patients did not experience any relapses during follow-up. Table 2 summarizes the data over follow-up, including numbers of PIRA, PIRMA, and purely cognitive PIRA events. It is worth noting that most of the PIRA events were “pure cognitive” events, with only 4 accompanied by a confirmed EDSS increase (none of the EDSS increases were due to the cerebral Functional System Score). None of the patients experienced more than one event in the same cognitive test.

**Table 2:** Follow-up data during the study

Median follow-up time in years (IQR)	5 (4.0–6.0)
Median number of visits per patient (IQR)	5.5 (5–6)
Patients with relapses during follow-up^a^, n (%)	14 (14.3%)
ARR during follow-up time, median (IQR)	0 (0–0)
Cognitive PIRA events	
Patients with at least one cognitive PIRA event at any test, n (%)^b^	36 (36.7%)
SDMT-PIRA, *n* events	11
VLMT-PIRA, *n* events	21
BVMT-PIRA, *n* events	10
Cognitive PIRMA events	
Patients with at least one cognitive PIRMA event at any test, *n* (%)	30 (30.6%)
SDMT-PIRMA, *n* events	7
VLMT-PIRMA, *n* events	17
BVMT-PIRMA, *n* events	9
Purely cognitive PIRA events	
Patients with at least one purely cognitive PIRA event at any test, *n* (%)	32 (32.7%)
Purely cognitive SDMT-PIRA, *n* events	9
Purely cognitive VLMT-PIRA, *n* events	20
Purely cognitive BVMT-PIRA, *n* events	9

^a^Two of the fourteen patients had two relapses during the follow-up time, while the rest had one relapse

^b^None of the patients experienced more than one event in the same cognitive test

Abbreviations: *ARR *annualized relapse rate, *BICAMS* Brief international cognitive assessment for MS, *BVMT* brief visuospatial Memory test, *IQR* interquartile range, *PIRA* progression independent of relapse activity, *PIRMA* progression independent of relapse and MRI activity, *SDMT* symbol-digit modalities test, *VLMT * Rey auditory verbal learning Test

**Fig. 2:**
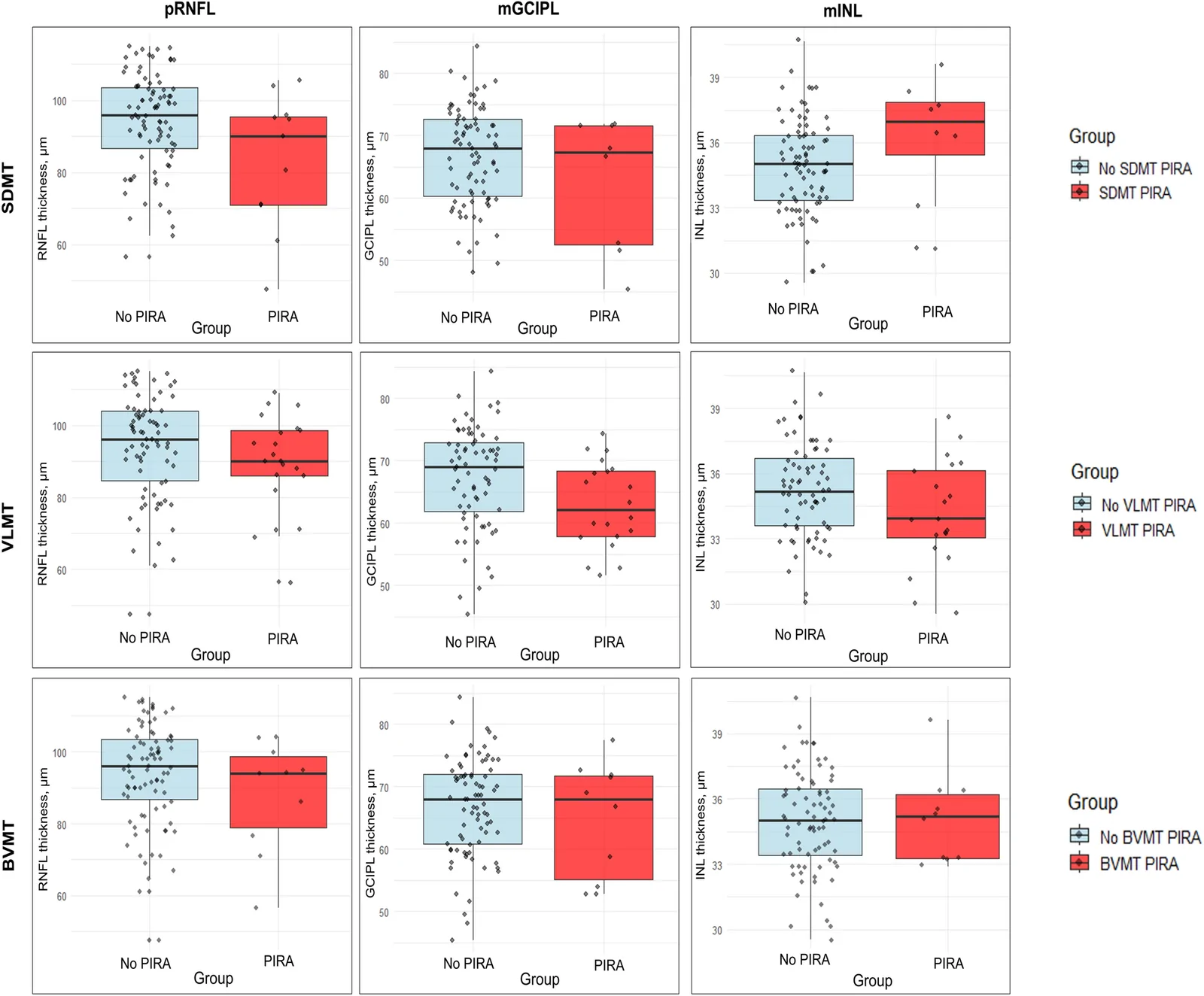
OCT measures at baseline in the PIRA versus no PIRA group for each BICAMS test. Abbreviations: *BICAMS *Brief International Cognitive Assessment for MS, *BVMT *brief visuospatial Memory test, *mGCIPL *macular ganglion cell-inner plexiform layers, *mINL *macular inner nuclear layer, *PIRA *progression independent of relapse activity, *pRNFL *peripapillary retinal nerve fiber layer, *SDMT *symbol-digit modalities test, *VLMT * Rey auditory verbal learning Test

Figure 2 illustrates baseline OCT metrics for patients with- vs. without PIRA events in each BICAMS test. The course of cognitive scores over time in both groups for each BICAMS test is shown as a figure in Online Resource 2.

### Primary endpoint: retinal layer thickness at baseline and risk of cognitive PIRA events

Each 1 μm increase in pRNFL thickness was associated with a 6% lower risk of SDMT-PIRA (hazard ratio (HR) 0.94, *p* = 0.027), while each 1 μm increase in mGCIPL thickness was associated with a 6% decrease in the risk of VLMT-PIRA events (HR 0.94, *p* = 0.040). Similarly, each 1 μm increase in mINL thickness was associated with a 20% lower risk of VLMT-PIRA (HR 0.80, *p* = 0.030). On the other hand, each 1 μm increase in mINL thickness was associated with a 50% *increase* in the risk of SDMT-PIRA (HR 1.50, *p* = 0.049).

**Fig. 3:**
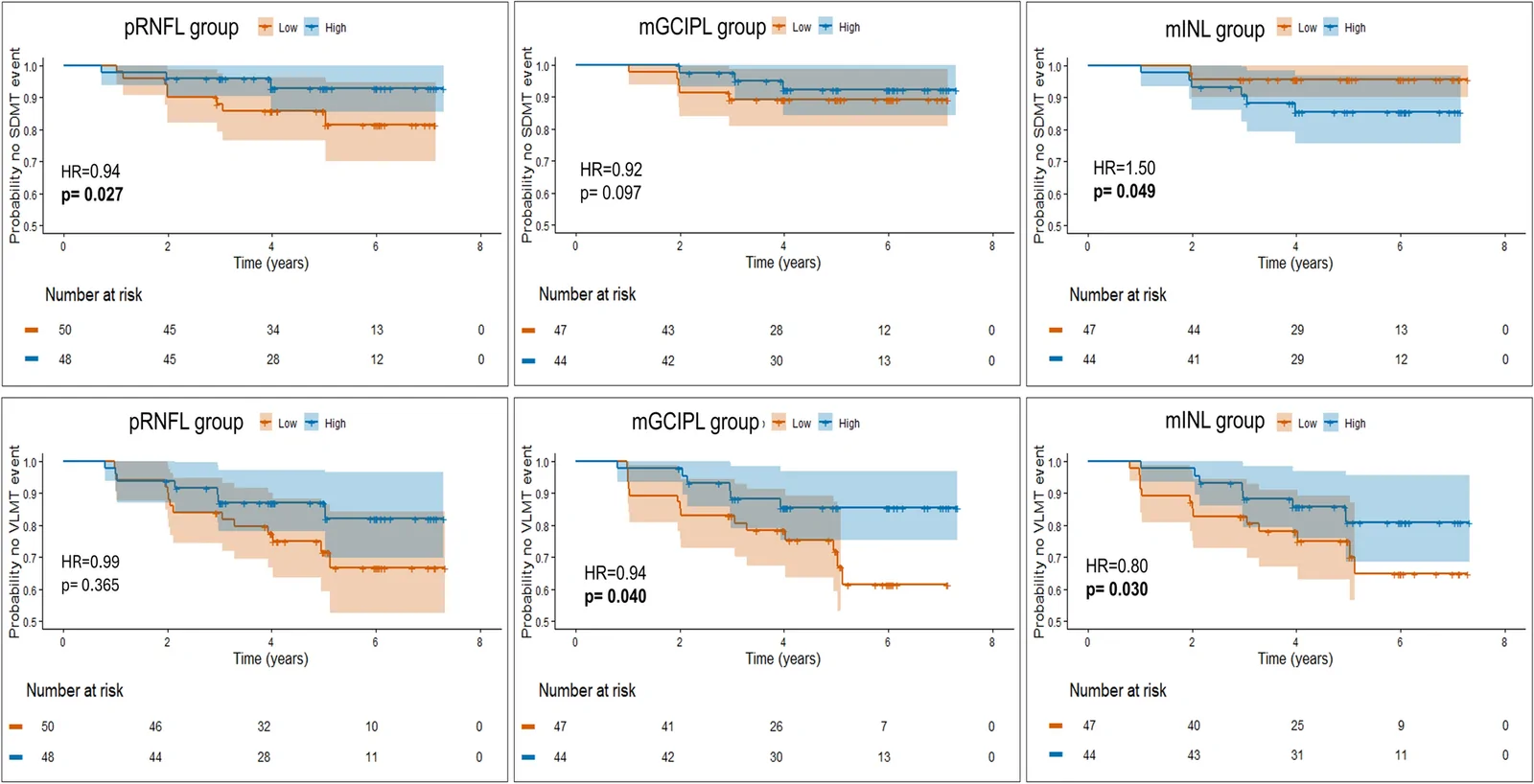
Kaplan–Meier curve for time to cognitive PIRA by thickness of different retinal layers. The groups “high” and “low” for each layer have been done using the median and for the purpose of visualization. Please note that the hazard ratio and *p* values showed in each figure correspond to the adjusted cox regression models where the thickness of each retinal layer is a continuous variable. Abbreviations: *HR *hazard ratio, *mGCIPL *macular ganglion cell-inner plexiform layers, *mINL * macular inner nuclear layer, *pRNFL *peripapillary retinal nerve fiber layer, *PIRA *progression independent of relapse activity, *SDMT * symbol-digit modalities test, *VLMT *Rey auditory verbal learning Test

**Fig. 4:**
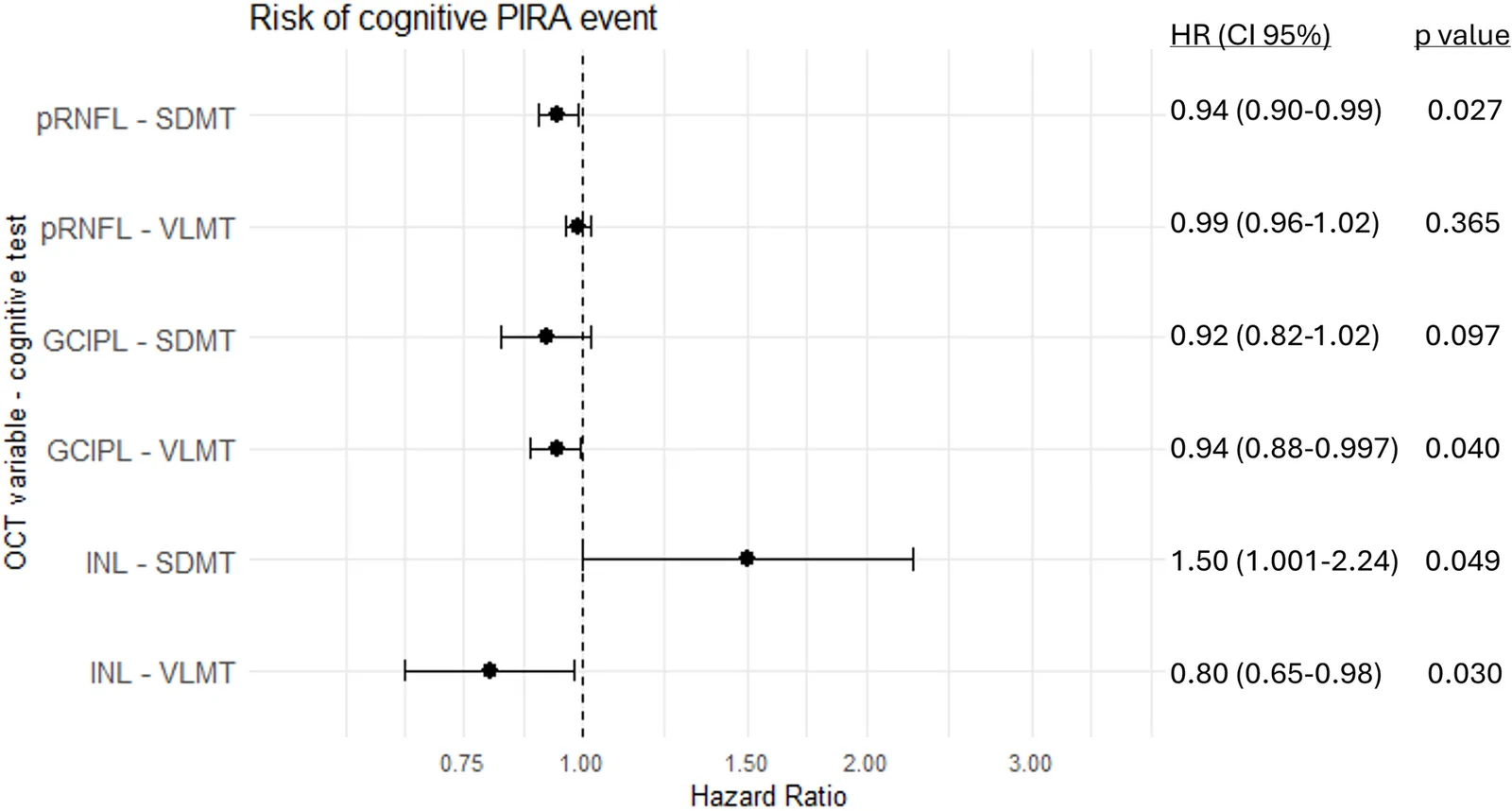
Forest plot of hazard ratios for cognitive PIRA by thickness of different retinal layers. Hazard ratios (HRs) and 95% confidence intervals (CI) for time to SDMT- and VLMT- PIRA events are shown for each predictor (baseline thickness of different retinal layers: pRNFL, mGCIPL, and mINL), deriving from the separate Cox regression models (each model including one retinal layer). Circles represent point estimates (hazard ratios), horizontal lines indicate 95% CI, and the vertical line represents the null effect (HR = 1). HRs represent the relative change in hazard of PIRA per unit-increase in the retinal layer thickness. Abbreviations: *CI * confidence interval, m*GCIPL *macular ganglion cell-inner plexiform layers, *HR * hazard ratio, m*INL * macular inner nuclear layer, *PIRA * progression independent of relapse activity, p*RNFL * peripapillary retinal nerve fiber layer, *SDMT * symbol-digit modalities test, *VLMT * Rey auditory verbal learning Test

These results (for all layers and tests) are summarized in Table 3 and depicted in Figs. 3 and 4.

**Table 3:** Summary of Cox regression models for retinal layer thickness at baseline and risk of cognitive PIRA-, cognitive PIRMA, and purely cognitive PIRA events over follow-up

Cognitive test	Retinal layer (*n* pat)	Cognitive PIRA			Cognitive PIRMA			Purely cognitive PIRA		
		*N* events	HR (95% CI)	*p*	*N* events	HR (95% CI)	*p*	*N* events	HR (95% CI)	*p*
SDMT	pRNFL (*n* = 97)	11	0.94 (0.90–0.99)	**0.027**	7	0.96 (0.90–1.02)	0.143	9	0.96 (0.91–1.01)	0.105
	mGCIPL (*n* = 90)	8	0.92 (0.82–1.02)	0.097	5	0.94 (0.82–1.07)	0.323	7	0.93 (0.83–1.04)	0.181
	mINL (*n* = 90)	8	1.50 (1.001–2.24)	**0.049**	5	2.64 (1.19–5.86)	**0.017**	7	1.60 (1.03–2.50)	**0.038**
VLMT	pRNFL (*n* = 97)	21	0.99 (0.96–1.02)	0.365	17	0.99 (0.96–1.03)	0.727	20	0.98 (0.95–1.01)	0.236
	mGCIPL (*n* = 90)	20	0.94 (0.88–0.997)	**0.040**	16	0.95 (0.89–1.02)	0.172	19	0.94 (0.88–0.999)	**0.050**
	mINL (*n* = 90)	20	0.80 (0.65–0.98)	**0.030**	16	0.72 (0.56–0.93)	**0.010**	19	0.79 (0.64–0.98)	**0.031**
BVMT	pRNFL (*n* = 96)	10	0.96 (0.91–1.02)	0.173	9	0.97 (0.92–1.02)	0.294	9	0.96 (0.91–1.01)	0.123
	mGCIPL (*n* = 89)	10	0.96 (0.87–1.06)	0.389	9	0.98 (0.89–1.09)	0.717	9	0.94 (0.85–1.04)	0.238
	mINL (*n* = 89)	10	1.11 (0.81–1.52)	0.521	9	1.20 (0.86–1.66)	0.283	9	1.07 (0.78–1.47)	0.687

All models were adjusted for age at baseline, years of education, baseline cognitive test score and dominant DMT group. Note that the models with macular layers have slightly lower number of patients/events, because more macular scans than ring scans had to be excluded

Abbreviations: *BICAMS *Brief international cognitive assessment for MS, *BVMT *brief visuospatial Memory test, *CI *confidence interval, *HR *hazard ratio, *mGCIPL *macular ganglion cell-inner plexiform layer; *mINL *macular inner nuclear layer, *pat * patients, *PIRA *progression independent of relapse activity; *PIRMA *progression independent of relapse and MRI activity, *pRNFL *peripapillary retinal nerve fiber layer, *SDMT *symbol-digit modalities test, *VLMT *Rey auditory verbal learning Test

Regarding the other independent variables in these models, there was an association between more years of education and higher risk of BVMT-PIRA events (HR 1.25–1.26, *p* = 0.041–0.043, slightly different in the three different models with each retinal layer). No other relationships were identified.

### Retinal layers at baseline and risk of cognitive PIRMA events

When investigating cognitive PIRMA events, we found overall similar results to the PIRA analysis (Table 3). The relationships between pRNFL and SDMT-PIRMA and between mGCIPL and VLMT-PIRMA were in the same direction as for PIRA (HR = 0.96 and 0.95 respectively) but not significant (Table 3). Of note, the number of PIRMA events was lower than PIRA (Table 3). Regarding mINL, Each 1 μm increase in thickness was associated with a 164% higher risk of SDMT-PIRMA (HR 2.64, *p* = 0.017), but at the same time with lower risk of VLMT-PIRMA events (HR 0.72, *p* = 0.010; Table 3).

### Retinal layers at baseline and risk of purely cognitive PIRA events

Ninety-one percent of cognitive PIRA events were purely cognitive, and the results of this analysis were overall very similar to the main analysis (Table 3). The relationship between pRNFL and SDMT-PIRA was in the same direction but not significant (HR 0.96, *p* = 0.105). The relationships between mGCIPL and VLMT-PIRA (HR 0.94, *p* = 0.050), as well as between mINL and SDMT-PIRA (HR 1.60, *p* = 0.038) and mINL and VLMT-PIRA (HR 0.79, *p* = 0.031) remained significant (Table 3). None of the covariates showed any associations in these purely cognitive PIRA models.

### Retinal layers at baseline and risk of cognitive PIRA, with adjustment for baseline MRI measures

The association between pRNFL and SDMT-PIRA persisted after adjustment for TLV (HR 0.93, *p* = 0.013) and for GMV (HR = 0.94, *p* = 0.030). The association between mGCIPL and VLMT-PIRA remained after adjustment for TLV (HR = 0.93, *p* = 0.033), but not for GMV (HR 0.94, *p* = 0.070). It should be noted that neither TLV nor GMV were associated with risk of cognitive PIRA in these models (for TLV: HR = 1 and for GMV HR = 1; *p* values slightly different in the different models but all > 0.05).

Regarding the mINL, the association between thicker layer and *increased* risk of SDMT-PIRA remained after adjustment for GMV (HR = 1.66, *p* = 0.025) but not for TLV (HR = 1.48, *p* = 0.065). The association between thicker layer and *decreased* risk of VLMT-PIRA persisted after adjustment for TLV and GMV (HR = 0.79, *p* = 0.036, and HR = 0.77, *p* = 0.020, respectively). Neither TLV nor GMV were associated with the risk of cognitive PIRA events in these models (HR for all = 1; all *p* > 0.05).

### Sensitivity analysis until year 5

The sensitivity analysis including only the cognitive data until year 5 (*n* = 98) showed overall very similar results to the main analysis (data not shown).

## Discussion

This is the first study to investigate the value of OCT in prognosticating the risk of cognitive PIRA in pwMS. Our main finding is that both thinner pRNFL and mGCIPL at BL were associated with increased risk of cognitive PIRA events: SDMT-PIRA for pRNFL and VLMT-PIRA for mGCIPL. Interestingly, the mINL displayed a different pattern, as a thinner layer prognosticated VLMT-PIRA, but thicker BL mINL was associated with increased risk of SDMT-PIRA. Importantly, the analysis was adjusted for age, educational level, and BL BICAMS scores, and thus, these results were not merely driven by older patients with cognitive impairment at BL. Moreover, the analysis of purely cognitive PIRA and PIRMA events showed overall similar results regarding the hazard ratios, while the *p* values varied, probably due to slight changes in total number of events.

Our findings are in line with the previous studies showing an association between OCT metrics and cognitive deterioration in pwMS [6, 11–13]. However, most of them used only the SDMT [6, 11, 12]. A single previous study employed the concept of PIRA [6], showing accelerated thinning of pRNFL and mGCIPL in patients with SDMT defined PIRA events over 4 years of follow-up [6], without examining the role of BL OCT metrics in risk assessment. To our knowledge, our study is the first to investigate the role of OCT metrics at BL in prognosticating the risk of cognitive PIRA using multidimensional cognitive testing (three BICAMS tests) in a well-characterized cohort with long follow-up (median of 5 years).

While PIRA was well described in the last years as the main driver of disability in MS [8], it is typically defined by physical impairment (based on the EDSS). However, the concept of cognitive PIRA was also proposed more recently [9]. Fuchs et al. observed that, in RRMS patients, PIRA accounts for most cognitive decline, compared to relapse-associated cognitive worsening. Furthermore, in their cohort, cognitive decline events occurred independently of EDSS worsening in more than half of the cases [9]. In our study, nearly all cognitive PIRA events were “purely cognitive” (91%). Fuchs et al. also concluded that cognitive PIRA is more likely to be captured through a battery of tests, such as BICAMS, compared to a single test, which is the approach we followed in our study. Interestingly, the number of PIRA events was highest for verbal memory (VLMT), followed by information processing speed (SDMT) in our patients.

While thinning of pRNFL and mGCIPL is well described in MS, the mINL exhibits a more complex and seemingly paradoxical behavior. mINL thickening has been associated with inflammatory (clinical and radiological) disease activity in RRMS, [22–24] and may decrease by immunotherapy [22]. On the other hand, postmortem analysis reveals neuronal loss in the mINL of MS eyes [25]. In line with this, a recent study [26] reported mINL *thinning* in PPMS and late phases of SPMS, but not in RRMS. In our study, thicker mINL was associated with a higher risk of cognitive SDMT-PIRA events, but at the same time, thinner mINL was associated with higher risk of VLMT-PIRA events. Our results suggest that a thicker mINL, potentially reflecting inflammatory processes and white matter (WM) damage in the brain [27], is associated with worsening in information processing speed, as measured by the SDMT. In contrast, thinner mINL, presumably driven by neurodegenerative mechanisms, appears to be more closely related to memory decline, as assessed by the VLMT. This interpretation is consistent with established associations between SDMT performance and WM damage in pwMS [28–31], given that delayed information processing is thought to be primarily driven by WM connectivity abnormalities [32, 33]. Conversely, memory impairment has been more strongly linked to neuronal damage, and verbal memory performance correlates with deep gray matter atrophy [34]. This aligns with the observed association between thinner mINL and memory decline in our cohort. However, it must be emphasized that no prior studies reported a relationship between mINL and cognitive deterioration in pwMS, and thus, our findings need to be interpreted with caution and replicated further. Moreover, the relationship between thicker mINL and SDMT-PIRA events remained in the sensitivity analysis using PIRMA, suggesting that it was not merely driven by MRI activity in our patients.

The models with adjustment for MRI parameters (TLV, GMV) showed overall interesting results. The relationship between pRNFL and SDMT-PIRA was independent of lesional and gray matter damage at BL; the same was true for the association between mINL and VLMT-PIRA. The association between mGCIPL and VLMT-PIRA did not remain after adjustment for GMV (*p* = 0.070), which may be due to the close relationship between loss of ganglion cells in the retina and gray matter loss in the brain [2]. Finally, the relationship between thicker mINL and increased risk of SDMT-PIRA did not remain after adjustment for lesional damage (TLV) at BL (*p* = 0.065), which may be due to the previous described association between thicker mINL and radiological disease activity [24]. It is worth noting that in all these models, the MRI parameters were not predictors of SDMT- or VLMT-PIRA, emphasizing the potentially added value of OCT metrics (as possibly more specific and more patient-friendly and highly reproducible technique [35]) to prognosticate cognitive worsening in pwMS.

Our study has limitations, like the relatively low number of pwMS with thus low number of total PIRA (and PIRMA) events and the further reduction of the participant number after year 5. However, our sensitivity analysis until year 5 showed very similar results with our main analysis. Furthermore, a possible learning effect should be considered, especially for the SDMT, where the same version was used through all visits. The potential impact of floor effects on detecting cognitive PIRA should be also mentioned; however, we do not believe that this played a major impact on our results, for the following reasons: We corrected for cognitive baseline scores in our analysis, the percentage of cognitively impaired patients at baseline was low and the threshold used to define the PIRA events was based on a percentage (10%), where the absolute change required becomes smaller as the score approaches the lower end of the scale. Finally, we did not account for neuropsychiatric comorbidities, fatigue, or sleep disturbances, all of which can influence cognitive performance. Key strengths of our study are the multidimensional cognitive testing in regular intervals in contrast to prior studies that investigated only SDMT [11, 12, 36], the relatively long follow-up compared to previous studies [12, 36, 37], and the inclusion of lesional and volumetric MRI data.

To conclude, our findings, which need further validation in larger cohorts, suggest that OCT can provide easily obtainable and noninvasive prognostically relevant biomarkers of progression in MS.

## Supplementary Information

Below is the link to the electronic supplementary material.

Supplementary file1 (PDF 196 KB)

Supplementary file2 (PDF 177 KB)

## Data Availability

The data of this study could be made available to qualified investigators upon reasonable request to the corresponding author.
