# Supplementary material for: Retinal layers prognosticate cognitive progression independent of relapse activity in multiple sclerosis
Source: J Neurol. 2026 Aug 6;273(9):517. doi: 10.1007/s00415-026-14036-0 (PMC13447463; doi:10.1007/s00415-026-14036-0)
Supplement: Supplementary file 1 [file 415_2026_14036_MOESM1_ESM.pdf]

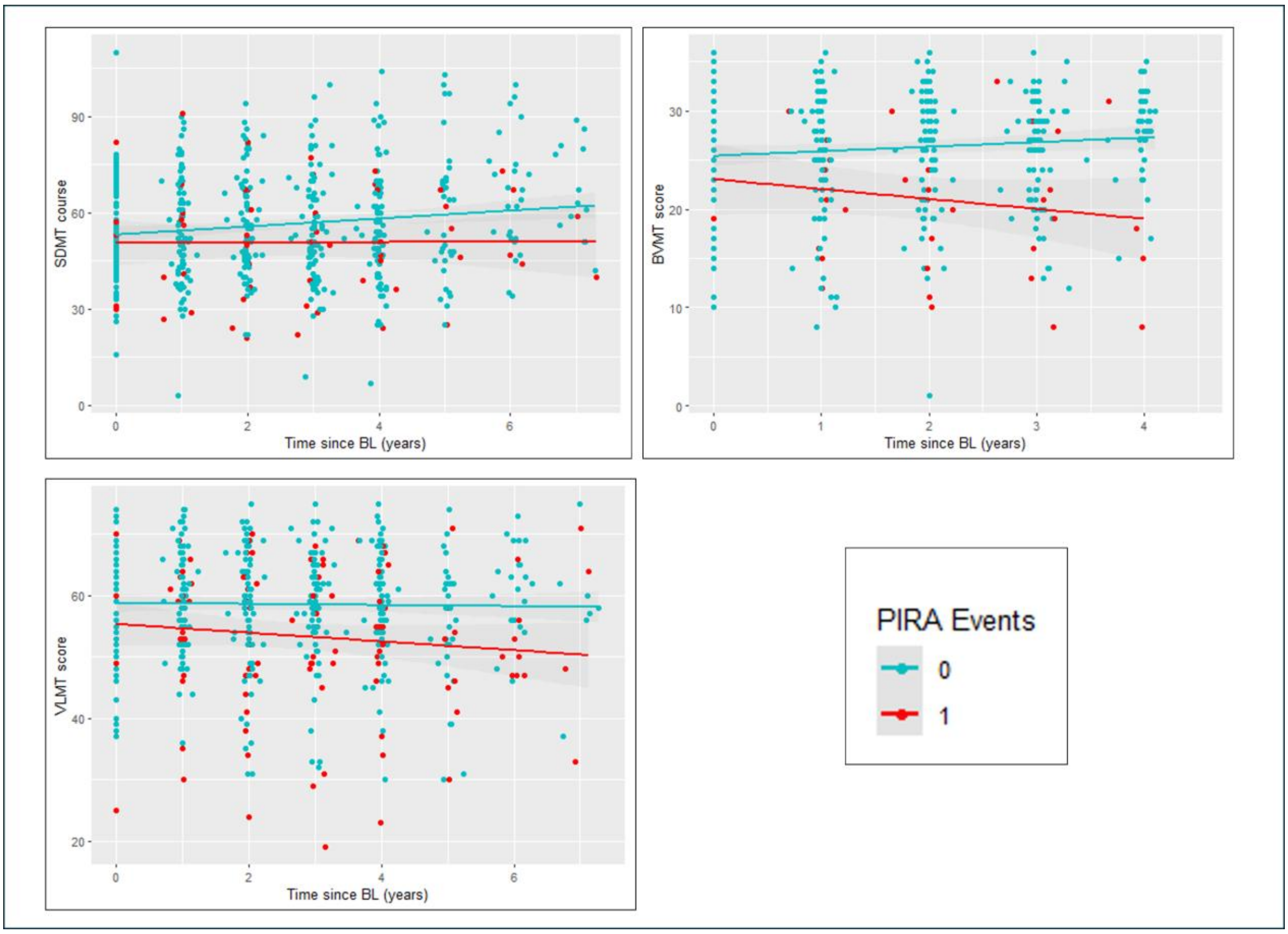


**Supplementary figure:** Cognitive scores over time in the PIRA versus no PIRA group for each BICAMS test.

The group without PIRA events in each test is shown in blue (PIRA events= 0). The group with PIRA events in each group is shown in red (PIRA events=1).

Abbreviations: BICAMS= Brief international cognitive assessment for MS; BL= baseline, BVMT= brief visuospatial Memory test; PIRA= progression independent of relapse activity; SDMT= symbol digit modalities test; VLMT= Rey auditory verbal learning Test
