# Supplementary material for: Retinal layers prognosticate cognitive progression independent of relapse activity in multiple sclerosis
Source: J Neurol. 2026 Aug 6;273(9):517. doi: 10.1007/s00415-026-14036-0 (PMC13447463; doi:10.1007/s00415-026-14036-0)
Supplement: Supplementary file 2 [file 415_2026_14036_MOESM2_ESM.pdf]

Supplementary table 1: Summary of the 9 APOSTEL 2.0 items regarding the Optical Coherence Tomography (OCT-) data of this study (1)**.**

Item		Description
1	Study protocol	Monocentric study in the Neurology Department of the University Hospital of Basel. The OCT examination had a duration of approximately 10-15 minutes and was usually performed on the same day as the assessment of the EDSS.<br><br>Study inclusion criteria: i) age ≥ 18 years old ii) MS diagnosis according to the 2010 revised McDonald criteria (2), and iii) eligibility for OCT (i.e. ability to fixate with each eye) and MRI (e.g. no claustrophobia).<br><br>Study exclusion criteria: i) presence of any retinal pathology that may interfere with the validity of the OCT, as previously described in the OSCAR-IB criteria (3), ii) refractive errors > 6 diopter, iii) history of both eyes being affected clinically by ON.
2	Acquisition device	Heidelberg Engineering Spectralis (Heidelberg, Germany)<br><br>Software version: Eye Explorer Heidelberg Engineering version 1.9.13.0<br><br>Device type: spectral domain (SD) OCT
3	Acquisition settings	Pupils were not pharmacologically dilated, but OCT was performed in a dark room. A single device was used, by three previously trained OCT-operators with excellent inter-rater agreement.

4	Scanning protocol	The scanning protocol included a circular scan around the optic disc and a volume scan from the macula centered on the fovea, both with the eye tracking function enabled.<br><br>i) The details of the circular (ring) scan for the assessment of the pRNFL thickness are: 3.4 mm ring scan, 12°, 1536 A-scans, ART between 12 and 100, high resolution (HR) setting, manual placement of the ring<br><br>ii) The details of the macular volume scan, for the assessment of the mGCIPL and mINL thicknesses were: 25°x30°, 61 vertically oriented B-scans, 768 A-scans per B-scan.
5	Fundoscopy	No fundus photography included in this study.
6	Post-acquisition data selection	Strict quality control of the OCT scans, using the validated OSCAR-IB criteria (3), which resulted in the exclusion of the following number of scans: peripapillary circular scans from 5 eyes (2 for quality reasons and 3 due to incidental findings) and macular volume scans from 21 eyes (13 due to quality reasons and 8 due to incidental findings).<br><br>Eye-selection strategy: Eyes with history of ON (n= 35) were excluded. For patients with unilateral ON, we included only the non-affected eye. Furthermore, we calculated the inter-eye asymmetry, using previously described thresholds: for pRNFL ≥5 μm and for mGCIPL ≥4 μm and excluding the eye with the lower thickness. In case of asymmetry only in pRNFL, we excluded only pRNFL of the worst eye, but not macular layers. In case of asymmetry in GCIPL, we excluded also INL of the worse eye, but not pRNFL thickness. For those patients without history of ON or ocular asymmetry, we took the average values of both eyes.

7	Post-acquisition analysis	Automated segmentation of the macular GCL, IPL and INL, as well as peripapillary RNFL using the software provided by the manufacturer (Eye Explorer 1.9.13.0, Heidelberg Engineering)<br><br>All scans were checked by two experienced OCT-raters and manually corrected where needed, especially in the outer ring area.<br><br>Mean thicknesses of mGCIPL and mINL were calculated using the 6-mm-diameter cylinder of the 1,3,6 mm ring adjacent to the fovea and transforming the volume to mean thickness according to the formula: thickness = volume / 0.02827433, where the constant derives from the cylindrical geometry of the ETDRS grid with a 1-, 3- and 6-mm diameter (radius = 3mm). For the combined mGCIPL volume, the volumes of the ganglion cell layer and the inner plexiform layer were added for each eye.
8	Nomenclature and abbreviations	Nomenclature compatible with Figure 1 in APOSTEL 2.0 guidelines:<br>- pRNFL: peripapillary retinal nerve fiber layer (µm)<br>- mGCIPL: macular ganglion cell- inner plexiform layer (µm)<br>- mINL: macular inner nuclear layer (µm)
9	Statistical approach	The analysis was performed per patient, due to the aim (association with cognitive PIRA, which is a per-patient event). Thus, we considered the average thicknesses of both eyes for the statistical analysis. However, in case of interocular asymmetry, we excluded the eye with the thinner layer, as described above. Furthermore, in case of unilateral ON, only the non-ON eye was considered. Cox regresion analysis was performed using R, as described in Methods.

Abbreviations: ART: automatic real-time tracking; EDSS: expanded disability status scale; mGCIPL: ganglion cell inner plexiform layer; mINL: inner nuclear layer; OCT: optical coherence tomography; ON: optic neuritis; PIRA: progression independent of relapse activity; pRNFL: peripapillary retinal nerve fiber layer.

References:

1. Aytulun A, Cruz-Herranz A, Aktas O, Balcer LJ, Balk L, Barboni P, et al. APOSTEL 2.0 Recommendations for Reporting Quantitative Optical Coherence Tomography Studies. Neurology. 2021 Jul 13;97(2):68–79.

2. Polman CH, Reingold SC, Banwell B, Clanet M, Cohen JA, Filippi M, et al. Diagnostic criteria for multiple sclerosis: 2010 Revisions to the McDonald criteria. Annals of Neurology. 2011;69(2):292–302.

3. Tewarie P, Balk L, Costello F, Green A, Martin R, Schippling S, et al. The OSCAR-IB Consensus Criteria for Retinal OCT Quality Assessment. PLOS ONE. 2012 Apr 19;7(4):e34823.

4. Nolan RC, Galetta SL, Frohman TC, Frohman EM, Calabresi PA, Castrillo-Viguera C, et al. Optimal Intereye Difference Thresholds in Retinal Nerve Fiber Layer Thickness for Predicting a Unilateral Optic Nerve Lesion in Multiple Sclerosis. J Neuro-Ophthalmol Off J North Am Neuro-Ophthalmol Soc. 2018 Dec;38(4):451–8.

5. Petzold A, Chua SYL, Khawaja AP, Keane PA, Khaw PT, Reisman C, et al. Retinal asymmetry in multiple sclerosis. Brain. 2021 Jan 1;144(1):224–35.
